# Purification and characterization of a cytochrome c with novel caspase-3 activation activity from the pathogenic fungus *Rhizopus arrhizus*

**DOI:** 10.1186/s12858-015-0050-9

**Published:** 2015-09-03

**Authors:** Manoj Saxena, Rohit Kumar Sharma, Josell Ramirez-Paz, Arthur D. Tinoco, Kai Griebenow

**Affiliations:** Department of Environmental Sciences, University of Puerto Rico, Rio Piedras Campus, P.O. Box 70377, San Juan, PR 00936-837 USA; Department of Chemistry, University of Puerto Rico, Rio Piedras Campus, P.O. Box 70377, San Juan, PR 00936-837 USA

## Abstract

**Background:**

Members of *Rhizopus* species are the most common cause of mucormycosis, a rare but often fatal fungal infection. Host induced pathogen apoptosis and pathogen induced host cell apoptosis are often involved in fungal infections. In many organisms, the release of mitochondrial cytochrome c can trigger apoptosis by activating caspase proteases, but the role of fungal cytochrome c in apoptosis remains unknown.

**Results:**

DNA sequence encoding *Rhizopus arrhizus* cytochrome c was cloned and expressed in *E. coli*. Both native and recombinant cytochrome c were purified using ion exchange followed by gel filtration chromatography. The identities of purified proteins were confirmed by MALDI-MS and UV-Visible spectroscopy. For the first time, we demonstrated that *Rhizopus arrhizus* cytochrome c could activate human capspase-3 in HeLa cell extracts. We also found that *Rhizopus arrhizus* cytochrome c has redox potential, peroxidase activity, and spectral properties similar to human and horse cytochrome c proteins.

**Conclusions:**

*Rhizopus arrhizus* cytochrome c can activate human caspase-3 in HeLa cell extracts and it possesses similar physical and spectral properties as human and horse cytochrome c. This protein was found to have a previously unknown potential to activate human caspase-3, an important step in the apoptosis cascade.

**Electronic supplementary material:**

The online version of this article (doi:10.1186/s12858-015-0050-9) contains supplementary material, which is available to authorized users.

## Background

Mucormycosis are rare but often life-threatening infections seen in immunocompromised, diabetic, and organ transplant patients [1]. These infections are difficult to treat and with an increase in the number of diabetic patients and organ transplants, in the future such infections are likely to increase [1, 2]. *Rhizopus* spp. accounts for the majority of the mucormycosis infections [1]. Results in recent decades suggest that apoptosis may play an important role in both establishment and clearance of fungal infections [3]. In response to fungal infections, the oxidative burst by host immune cells could help in infection clearance by triggering apoptosis in the fungus [4, 5]. Also, pathogen-induced apoptosis in host immune cells could help the pathogen to establish the infection [6]. Thus, the success of an infection partly depends on the outcome of such host pathogen interactions. It has been shown that extracts from the filamentous fungus could induce apoptosis in human cells [6, 7], but the identity of the protein/factor responsible for the induction of apoptosis remains unknown. In recent decades, it has also been established that in many organisms cyt c plays an important role in apoptosis [8, 9]. During apoptosis, cyt c is released from the mitochondria into the cytoplasm and binds to apoptosis protease-activating factor-1 (Apaf-1) thus triggering a caspase activation cascade [8]. For example, in humans, cyt c can activate caspase-3 in cell-free activation assay [9]. The first evidence of the existence of apoptosis in fungi came from the study by Medeo et al. of a *Saccharomyces cerevisiae* mutant that showed signs of apoptosis [10]. Since then, many homologous mammalian apoptotic proteins were discovered in budding yeast [11]. In an important finding, using deletion mutants which were unable to produce functional cyt c, Silva et al. presented the first evidence that cyt c is involved in hyperosmotic stress induced yeast apoptosis [12]. However, the nature of the role cyt c plays in apoptosis of filamentous fungi like *R. arrhizus* remains unknown.

To characterize *R. arrhizus* cyt c, we cloned the gene for cyt c from *R. arrhizus* in *E. coli*. The purified cyt c was compared with two mammalian cyt c (horse and human). We selected *R. arrhizus* cyt c as a candidate for this study mainly for two reasons. First, this fungal cyt c is distantly related to mammalian cyt c and its biochemical properties are largely unknown.

Secondly, *R. arrhizus* is a pathogenic fungus and any information gained on the pro-apoptotic activity of cyt c may also contribute to the identification of better therapeutic targets. We found that cyt c of *R. arrhizus* has similar biochemical properties to mammalian cyt c.

## Results and discussion

**Fig. 1:**
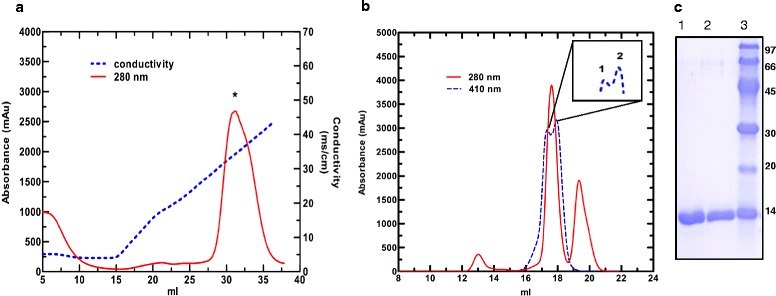
**a** Elution profile of recombinant *R. arrhizus* cyt c on a Hi-Trap SP Sepharose column (5 ml). The bound protein was eluted with an increasing NaCl gradient over 7 column volumes. Peak fractions containing cyt c protein is indicated by *. **b** Elution profile of the pooled HiTrap SP Sepharose peak fractions loaded onto a Superdex 200 GL column. Cyt c elution was followed using 410 nm absorption. Cyt c protein elutes as two unresolved peaks (in *inset peak 1* and *2*). **c** Coomassie stained 15 % SDS-PAGE gel loaded (*lane 1* and *2*) with the *peak 1* and *2* from the Superdex 200 GL column, *lane 3* = marker proteins. Cyt c band moves close to the 14 kDa marker band

A 19-fold purification was achieved for recombinant *R. arrhizus* cyt c using a three step process involving ammonium sulphate precipitation, cation exchange and gel filtration chromatography (Fig. 1, Table 1). The purified recombinant cyt c protein eluted from gel filtration column with absorption ratio 410/280 nm of >4.0 (Fig. 1b, Table 1). Purity was also checked by SDS electrophoresis. The eluted cyt c produced one band, which had an apparent MW of ca. 14 kDa (Fig. 1c). Since this is the first report on purification of recombinant *R. arrhizus* cyt c, we compared our purification results with those reported by others using similar plasmids. We obtained ~9 mg of cyt c with a 410/280 ratio of >4 from 1 L of *E. coli* culture. Our yield was similar to the reported yield for human cyt c of >8 mg L^−1^ [13]. Patel et al. reported a yield of ~15 mg L^−1^ for horse cyt c [14]. However, in that work the authors only performed a two-step purification (ammonium sulphate precipitation and a cation exchange column) [14]. Our yield was only marginally lower if we compare our yield after the second purification step (Table 1).

**Table 1:** Purification of recombinant *R. arrhizus* cyt c

Purification step	Total Cyt c (mg)	Purity (OD410/280)	Recovery (%)	Fold purification
Lysate	43.9 ± 1.2	0.223 ± 0.005	100	1.00
Ammonium Sulphate (Precipitation)	30.8 ± 0.7	0.306 ± 0.001	70.2	1.37
Dialysis	20.7 ± 0.2	0.440 ± 0.001	47.2	1.97
Cation exchange	12.4 ± 0.3	3.91 ± 0.10	28.2	17.50
Gel filtration	9.12 ± 0.13	4.26 ± 0.03	19.7	19.10

**Fig. 2:**
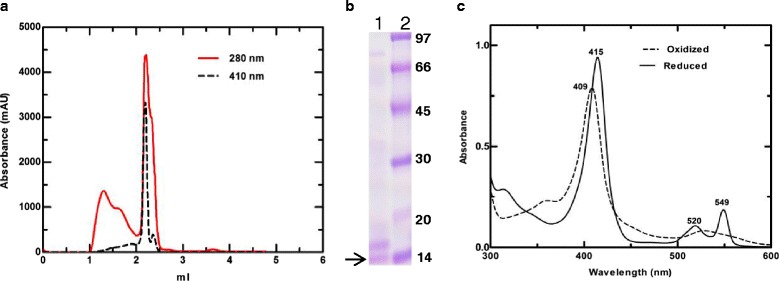
**a** Elution profile of native *R. arrhizus* cyt c on a Superdex 75 column. **b** 15 % SDS PAGE gel showing the peak fraction (*lane*-*1*), Molecular weight marker (*lane*-*2*). Band marked with an *arrow* indicate the position of protein band identified as cyt c. **c** Absorption spectra of the oxidized (---) and reduced form (^_^) of the native *R. arrhizus* cyt c. The oxidized form shows a peak in the Soret region at 409 nm while in the reduced form the Soret peak shifts to 415 nm

In the case of the native *R. arrhizus* cyt c (protein isolated form commercial preparation of lipase) the final protein eluted from a Superdex 75 column as a single peak (Fig. 2a), but the 410/280 nm ratio was only 0.65. Efforts to further purify the native protein using an additional anion exchange column to trap impurities did not result in any improvement.

**Fig. 3:**
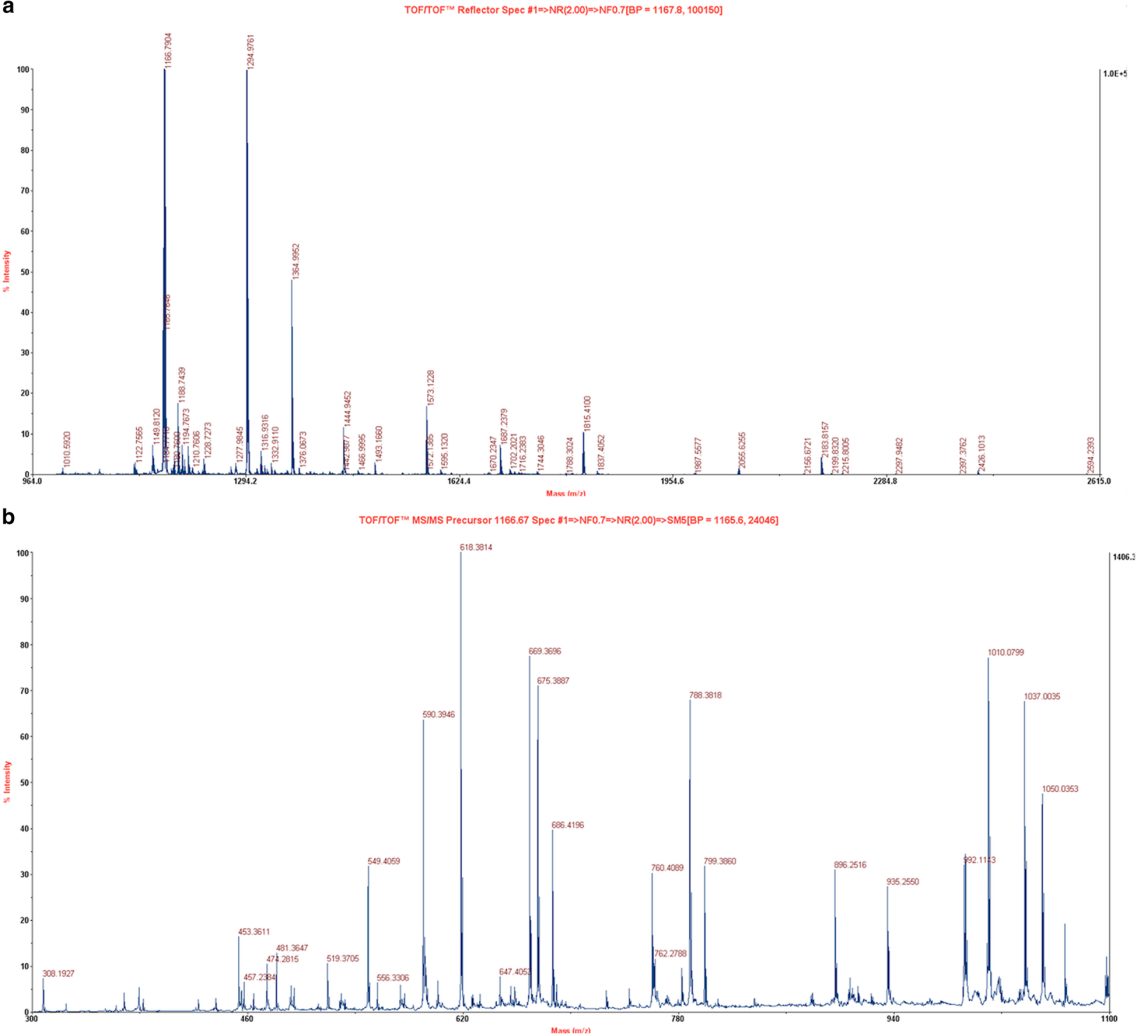
MS spectra of the recombinant Rhizopus cyt c and MS/MS spectra of native Rhizopus cyt c collected on a MALDI TOF/TOF instrument. **a** MS spectra of the recombinant protein digested with trypsin. **b** MS/MS of precursor peptide (1166.67 Da) from the native *R. arrhizus* cyt c protein digested with trypsin. Mascot online server was used to confirm the identity of both native and the recombinant proteins

This concurs with a previous study that reported difficulties in purifying this protein from *R. oryzae* (syn. to *R. arrhizus*) to high purity [15, 16]. The relative molecular weight determined for the native *R. arrhizus* cyt c by SDS-PAGE was 12.55 ± 1.27 kDa (Fig. 2b) in agreement with expectations for its molecular weight. Identity of both, the native and the recombinant protein bands was unequivocally confirmed by MALDI tandem mass spectroscopy (Fig. 3 and Additional file 1: Figure S1).

The UV–vis spectra of the oxidized native *R. arrhizus* cyt c show peaks at 409 and 529 nm and in the reduced state, α and β peaks are prominent at 549 and 520 nm, respectively (Fig. 2c). Similar values were seen for the recombinant *R. arrhizus* cyt c. These values are close to other type-c cytochromes, thus suggesting a similar heme environment (Table 2).

**Table 2:** Comparison of the spectral properties of horse, human and *R. arrhizus* cyt c

Cyt c	α_max_ (nm)	β_max_ (nm)	α/β	Charge transfer band (nm)
Horse	550	521	1.87	695
Human	549	520	2.86	695
nRhizopus	549	520	1.87	700
rRhizopus	549	520	1.87	699

nRhizopus: native cyt c from *R. arrhizus*; rRhizopus: recombinant cyt c from *R. arrhizus*

The presence of an absorption maximum at ~700 nm in both recombinant and the native *R. arrhizus* ferri cytochrome (Additional file 2: Figure S2A & B) suggests that, like in other c-type cytochromes, methionine is one of the axial heme ligands. Additionally, sequence alignment shows that residues corresponding to Met-80 and His-18 are conserved in 14 fungal cyt c [17], as well as in human and horse cyt c.

**Fig. 4:**
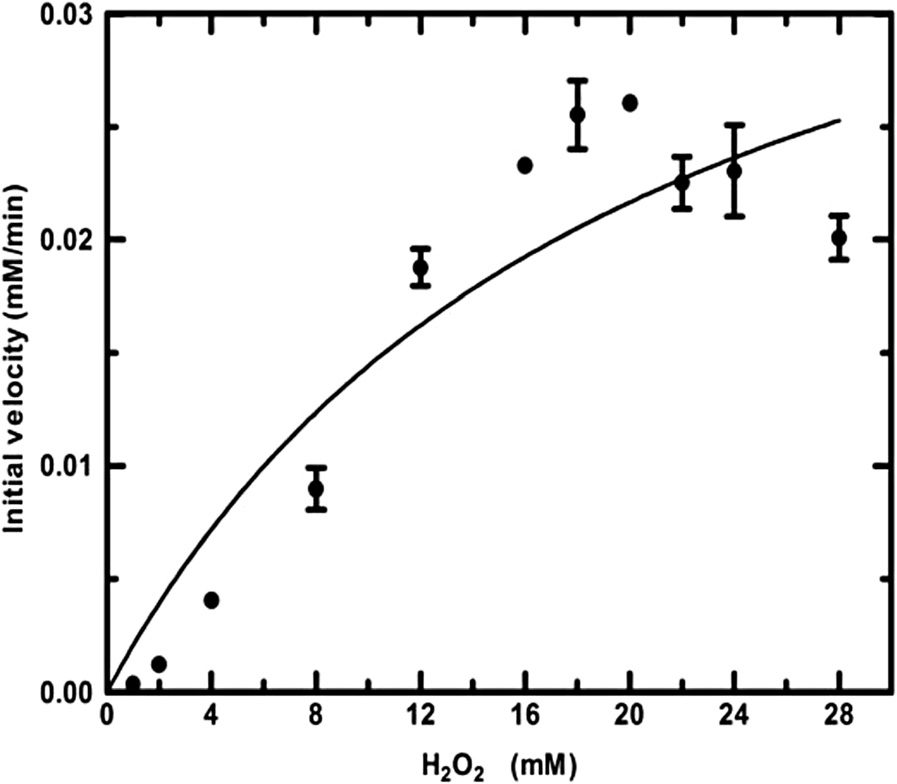
Peroxidase activity of native *R. arrhizus* cyt c as a function of substrate (H_2_O_2_) concentration. Shown here is the amount of oxidized ABTS (y-axis) formed per minute at different H_2_O_2_ concentrations (X-axis). *Error bars* represent the standard deviation

The redox potential of native and recombinant *R. arrhizus* cyt c was found to be similar to that of human and horse cyt c, respectively, with no statistically significant difference (Fig. 4 and Additional file 3: Figure S3). The redox potential measurements for human and horse cyt c with 263.43 and 268.64 mV, respectively, were in close agreement with earlier reported values validating our technique [18, 19]. The redox potential measurements of native and recombinant *R. arrhizus* cyt c were 266.90 and 270.04 mV and statistically the same. These results further support structural similarity between the recombinant and native *R. arrhizus* cyt c proteins.

A major portion of cyt c remains loosely associated with the inner mitochondrial membrane via ionic interactions with the negatively charged mitochondrion-specific phospholipid, cardiolipin. Kagan et al. had shown that cardiolipin specific peroxidase activity of cardiolipin bound cyt c may play an important role in triggering apoptosis by aiding in the release of pro-apoptotic proteins, including cyt c, from the matrix of the mitochondria [20]. Furthermore, peroxidase activity assay provides an indirect measure of heme accessibility and we therefore compared the peroxidase activities of mammalian and *R. arrhizus* cyt c.

The peroxidase activity of native *R. arrhizus* cyt c was found to be low compared to other known peroxidases, which is a general characteristic of type-c cytochromes. Recombinant cyt c showed a higher K_cat_ and V_max_ compared to native *R. arrhizus* cyt c. These variations are most likely due to the higher purity of the recombinant protein compared to native one (Table 2, Table 3). Also, a high peroxidase activity could be related to partial denaturation and thus a more accessible heme in the non-recombinant cyt c. A partial denaturation can involve the breaking of the methionine coordination that leaves the heme more accessible for catalysis [21]. However, this is ruled out by the UV-spectra of the recombinant cyt c because they show the presence of the charge transfer band thus indicating that methionine coordination is intact (Additional file 2: Figure S2).

**Table 3:** Kinetic parameters for the peroxidase activity of horse, human and *R. arrhizus* cyt c

Cyt c	K_M_ (M)	V_max_ (mM min^−1^)	K_cat_ (min^−1^)
Horse	23.0 ± 6.5	0.0541 ± 0.0091	22.0 ± 4.0
Human	3.74 ± 0.80	0.0190 ± 0.0020	7.60 ± 0.62
nRhizopus	20.1 ± 8.3	0.0434 ± 0.0093	29.0 ± 6.2
rRhizopus	13.12 ± 5.05	0.0892 ± 0.0203	59.47 ± 13.55

Results are the means ± S.D. of three readings

nRhizopus: native cyt c from *R. arrhizus*; rRhizopus: recombinant cyt c from *R. arrhizus*

Native *R. arrhizus* cyt c showed similar activity as horse cyt c while it was higher than human cyt c (Table 3). On the other hand, judging from K_M_ values the affinity of *R. arrhizus* cyt c seems to be lower than human cyt c, and very similar compared to horse cyt c (Table 3). The K_M_ value measured for horse cyt c was in close agreement with Kim et al. [22] but varied from the one reported by Randi et al. [23]. It is important to note that we used non-linear regression to analyze the kinetics while the two cited studies used Lineweaver-Burk plots, a method considered less reliable [24].

We noted the presence of the cyt c in good amounts in a commercial preparation of a secreted lipase of *R. arrhizus* (Fig. 2). Interestingly, in an earlier study, the authors have indicated the presence of a “soluble factor” in *R. arrhizus* extract that can activate caspase, but they did not identify this factor [7]. Based on these observations, we tried to verify whether this unknown water-soluble factor could be cyt c present in the supernatant of the *R. arrhizus* cultures or not. Efforts to locate the cyt c in supernatant fractions with heme staining (data not shown) and using an antibody against cyt c were not successful (Additional file 4: Figure S4). These results suggest that under our test conditions, the protein is not secreted into the supernatant. However, the possibilities that the protein might be secreted in response to some special cue, or in trace amounts, could not be ruled out.

**Fig. 5:**
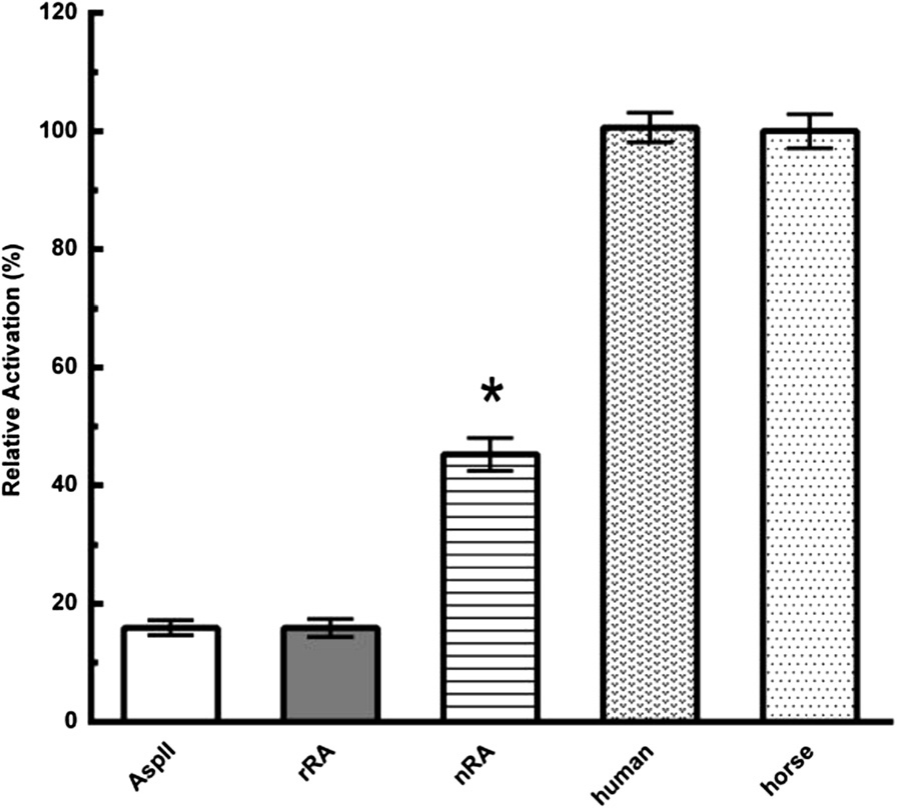
Comparison of cell-free caspase-3 activation by horse, human and R. arrhizus cyt c. Caspase-3 activation was followed at 405 nm and normalized to the horse cyt c signal. rRA represents the recombinant and nRA the native cyt c from R. arrhizus, while AspII represents the negative control contains asparaginase-II (no cyt c). Each column represents the mean of independent measurements, with *error bars* representing the standard deviation. The initial incubation to activate caspase was performed with samples at 10 μM final concentration. The native cyt c signal (*) was significantly different from the others (*P* < 0.05)

In the cell free caspase-3 activation assay, compared to all cyt c tested, the recombinant *R. arrhizus* cyt c showed the lowest activity. Its activity was similar to the negative control, a cell lysate with asparaginase-II (Fig. 5). The native *R. arrhizus* cyt c showed a statistically significant (*P* < 0.05) higher caspase-3 signal but under the same conditions no statistically significant difference was observed in the signals of recombinant *R. arrhizus* cyt c and the negative asparaginase-II control (Fig. 5).

In our *in vitro* caspase-3 assay, we did not observe any caspase-3 activity in the aqueous extracts prepared from the *R. arrhizus* culture (Additional file 5: Figure S5). This result indicate that the source of previously reported activity in the aqueous extract is unlikely to be cyt c [7].

Compared to recombinant cyt c, higher caspase-3 activation by native *R. arrhizus* cyt c could be due to the presence of other unidentified factor/s. Identity of these factors is a matter of further investigation and beyond the scope of the present study.

**Fig. 6:**
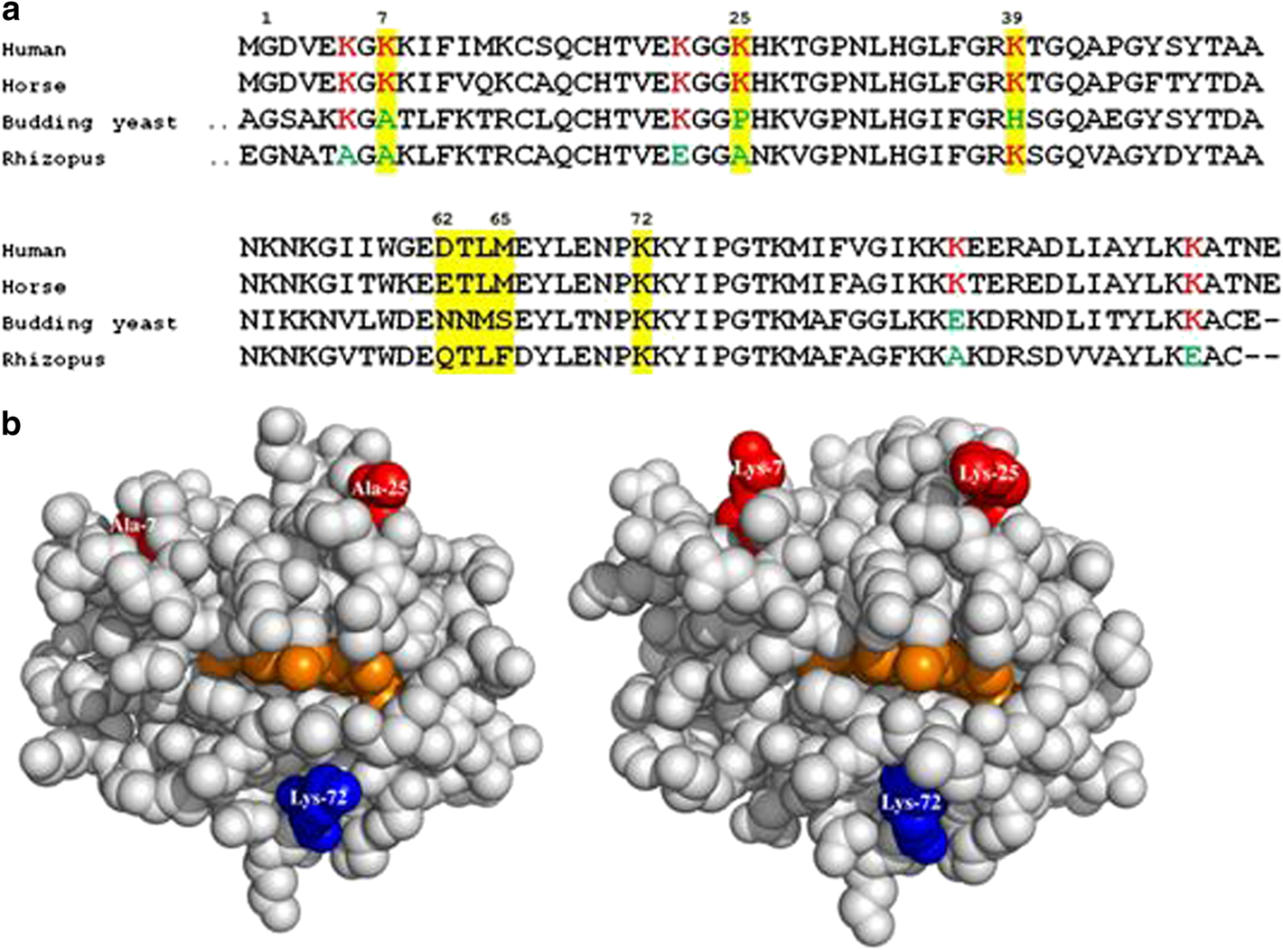
**a** Sequence alignment of *R. arrhizus* cyt c with human, horse, and budding yeast cyt c. The *shaded* areas are the residues (in horse) essentially involved in Apaf-1 binding. Residues in *R. arrhizus* showing charge reversal/neutralization (with respect to horse Lys) substitutions are shown in *green*. The numbering position of residues is with respect to horse cyt c. **b** Protein models of *R. arrhizus* and horse cyt c showing positions of 3 important lysine residues involved in Apaf-1 binding. From *left* to *right*: *R. arrhizus* model (generated using Swiss-model homology modeling server) and horse cyt c (PDB ID-1HRC). In *R. arrhizus*, two residues important for Apaf-1 binding, lysine 7 and 25 (*red*) are substituted by alanine. Another important lysine residue (*blue*) is conserved in both *R. arrhizus* and horse cyt c. Both images were generated using PyMOL

Additionally, the activation differences between native *R. arrhizus* and the horse cyt c could be due to the variations in distribution of positive patches on the *R. arrhizus* cyt c surface, which are known to be critical for cyt c/Apaf-1 interaction. Cyt c and Apaf-1 interact through an extensive region. Many lysine residues (7, 8, 25, 39 and 72) spread across the surface of cyt c are known to be important for this interaction. Disruption of these residues by site directed mutagenesis has been shown to abolish or lower the ability of horse and human cytochrome to activate caspases [25, 26]. In the case of native *R. arrhizus*, the reduction in caspase-3 activation was seen when compared to horse cyt c. Sequence comparison with horse cyt c revealed that in the native *R. arrhizus* cyt c two of these lysine residues (7 and 25) are occupied by alanine (Fig. 6). We propose that these changes to hydrophobic non-polar groups (Ala) could decrease cyt c affinity for Apaf-1. The lower caspase-3 activation by the native *R. arrhizus* cyt c is a potential reflection of this effect. Indeed, these two lysine residues (7 and 25) were demonstrated to be essential for Apaf-1 binding, since a 10–100 fold drop in caspase activity was observed by Yu et al. when these amino acids were mutated in combination with other Apaf-1 interacting residues in horse cyt c [25].

In *Saccharomyces cerevisiae cyt c*, a tri-methylation at lysine 72 was attributed for being responsible for its lower caspase activation potential [26]. We reasoned that it is possible that a similar tri-methylation on the K72 in *R. arrhizus* cyt c could also partly account for its lower caspase activity as seen in our assays (Fig. 5). However, our MALDI data does not support the presence of Lys-72 methylation (Additional file 6: Figure S6).

We also found four additional lysine substitutions in *R. arrhizus* cyt c (K5A, K22E, K88A & K100E) that have not been studied before, and could potentially contribute to its reduced caspase-3 activation (Fig. 6). It will be interesting to see if an *R. arrhizus* mutant with A7K and A25K mutations shows increased caspase-3 activation to the level similar to that of horse cyt c, since this would help to elucidate the role of other lysine substitutions seen in *R. arrhizus* cyt c.

## Conclusions

We have reported here a comparative characterization of *R. arrhizus* cyt c. To the best of our knowledge, this is the first report on recombinant purification and biochemical characterization of the *R. arrhizus* cyt c.

The mitochondrial proteins are attractive targets for new antifungal drugs [27]. A better understanding of the role of specific sequences in cyt c’s ability to induce apoptosis and its differences with mammalian cyt c could lead to the identification of methods for exclusive targeting of this fungal pathogen. It will be interesting to see if the amino acid differences in cyt c of *R. arrhizus* could provide it with sufficient specificity to induce apoptosis exclusively in fungal cells. If this turns out to be the case, designing of fungi specific apoptosis inducing drugs may be possible. Although results of targeting human cancer cells by actively inducing apoptosis using mammalian cyt c delivery have been promising [28, 29], replicating them in the fungal cells could be very challenging due to the presence of their cell wall. Our results show the ability of *R. arrhizus* cyt c to activate caspase-3. This finding indicates that this pathogen could potentially use a similar mechanism in vivo to establish infections. To explore the possibility of existence of such mechanism in vivo, a study of clinical isolates and samples from infected patients would be helpful. At present such studies are impeded by the lack of our knowledge about the role of *R. arrhizus* cyt c in apoptosis. This study will help catalyze exploration into the possible involvement of cyt c in establishing mucormycosis infections.

## Methods

### Gene cloning and protein purification

Recombinant *R. arrhizus* cyt c was expressed from a construct made by modifying the pBTR1 plasmid [13]. pBTR1 was a gift from Gary Pielak (Addgene plasmid # 22468). It contained human cyt c gene and a heme lyase gene from *Saccharomyces cerevisiae*. The lyase gene is essential for heme incorporation into cyt c protein. In the modified plasmid (pBRA), human cyt c was replaced with the gene encoding *R. arrhizus* cyt c. The sequence coding for *R. arrhizus* cyt c was commercially synthesized (GenScript) and ligated into pBTR1 vector (without the human cyt c gene) using Gibson Assembly (NEB). The primers utilized and steps performed to obtain pBRA are provided in the Additional file (primer and vector details are provided in Additional file 7: Figure S7). pBRA was transformed into BL21(DE3)T1^R^ cells (Sigma). The bacterial cultures were grown in TB media (12 gmL^−1^ tryptone, 24 gmL^−1^ yeast extract, 8 mL glycerol, 2.3 gL^−1^ KH_2_PO_4_ and 12.5 gL^−1^ K_2_HPO_4_) containing ampicillin (100 mgL^−1^) at 37 °C under constant shaking at 220 rpm. A 5 mL overnight culture grown from a single colony was used to inoculate a 500 mL culture. The culture was harvested after 18 h of growth at 37 °C by centrifuging at 7000 g at 4 °C. The pellet obtained was incubated with lysozyme 3 gL^−1^ and 3 mg of DNase 1 in lysis buffer (50 mM Tris-Cl pH 6.8 and 1 mM EDTA) for 8 h at 4 °C with stirring. The lysis mixture was sonicated on ice for 5 min. Lysed cells were centrifuged at 7000 g on a Sorvall RC 6 plus (Thermo Scientific) at 4 °C. Ammonium sulfate was added slowly to the supernatant at 4 °C under constant stirring to a final concentration of 350 gL^−1^. The resulting precipitate was centrifuged at 7000 g for 30 min at 4 °C. The supernatant containing cyt c was dialyzed using a 3.5 kDa dialysis membrane overnight against 20 mM sodium phosphate buffer at pH 6.8. Dialyzed protein was loaded on a HiTrap SP Sepharose column (GE Healthcare) equilibrated with buffer A (40 mM sodium phosphate buffer at pH 6.8). Bound protein was eluted with elution buffer B (40 mM sodium phosphate at pH 6.8 and 1 M NaCl) using a linear gradient from 0 % B to 100 % B over a 7 column volume. Peak fractions with an absorbance ratio of 410/280 nm > 2.5 were pooled and loaded on a Superdex 200 gel filtration column (GE Healthcare). Protein elution from the Superdex 200 column was followed by absorption at 410 and 280 nm. Peak fractions were analyzed by loading onto a 15 % SDS-PAGE. Native *R. arrhizus* cyt c was purified using lipase of *R. arrhizus* obtained from Sigma using a similar scheme as that of the recombinant protein.

Human and horse cyt c were used as controls in all the assays performed unless otherwise stated. Human cyt c was expressed in *E. coli* using plasmid pBRT1 and purification was done as described by Olteanu et al. [13]. Horse heart cyt c was purchased from Sigma and used without further purification.

### UV–vis spectroscopy

All spectra were measured using a UV–Vis NanoDrop 2000/2000c (Thermo Scientific) spectrophotometer except for the charge transfer band (CTB). The CTB region was measured on a UV-2450 Shimadzu spectrophotometer, with a slit width of 0.5 nm. The peak location was determined by using the picking algorithm of the UV-Probe 2.33 software (Shimadzu).

### Trypsin digestion and mass spectrometry

In-gel trypsin digestion was performed using 500–1000 ng of both native and recombinant *R. arrhizus* cyt c as described [30]. In brief, the gel-eluted peptides resulting from trypsin digestion were first vacuum dried and later purified on a C18 reverse phase tip column (Millipore) using the instructions supplied by the manufacturer. Trypsin-digested peptides were eluted in a total volume of 5 μl of acetonitrile and 0.1 % trifluoro acetic acid solution (50:50 *v*/*v*). The purified peptides were directly spotted onto the MALDI plate following the dried droplet method [31]. The matrix solution used contained 5 mg/mL α-cyano-4-hydrooxycinnamic acid (HCCA, Sigma) in acetonitrile and 0.1 % trifluoro acetic acid (50:50 *v*/*v*). Tandem mass spectrometric analysis was performed using an ABSCIEX 4800 Plus MALDI TOF/TOF™ Analyzer in Top 6 mode. The spectra were collected in positive ion reflector mode with 500–1000 laser shots per spectrum. Protein identification was performed using the Mascot server (Matrix Science, http://www.matrixscience.com/search_form_select.html).

### Redox potential

The redox potential of *R. arrhizus cyt* c was determined as described [18] with some minor modifications. Briefly, 100 mM stock solutions of potassium ferrocyanide (ferroCN), potassium ferricyanide (ferriCN), and (+)-sodium L-ascorbate were prepared in 100 mM potassium phosphate buffer at pH 7.0. Prior to use, all solutions were vacuum degasified and purged with nitrogen for 2–3 min. The absorbance of samples was recorded at 550 nm using a NanoDrop 2000/2000c spectrophotometer (Thermo Scientific). A plot of log([ferroCN]/[ferriCN]) vs. log([ferrocyt c]/[ferricyt c]) was drawn using Prism 5 (GraphPad Software).

### Peroxidase assay

Cyt c peroxidase activity was measured as described [22]. Briefly, each reaction was performed in a total reaction volume of 150 μL with 50 μM 2,2′-azino-bis(3-ethylbenzothiazoline-6-sulphonic acid) (ABTS), 2.5 μM for human and horse cyt c, and 1.5 μM for native *R. arrhizus* cyt c. The concentration of cyt c was determined using the absorption coefficient of 29.5 mM^−1^ cm^−1^ at 550 nm [32]. Each reaction was followed for 40 s by recording the absorption of oxidized ABTS at 405 nm. The concentration of oxidized ABTS was calculated using the extinction coefficient of 36.8 mM^−1^ cm^−1^ at 405 nm. For each reaction with varying H_2_O_2_ concentrations (ranging from 1.0 to 28 mM), initial rates were measured using the linear part of the absorption curve. All absorption readings were made using a Shimadzu UV-2450 spectrophotometer using a cell of 1 cm path length at 25 °C. The Michaelis-Menten constants K_M_, K_cat_ and V_max_ were determined by nonlinear regression curve fitting using Prism 5 (GraphPad Software).

### Cell free caspase-3 activation assay

HeLa cells were grown to 90 % confluency, harvested, and disrupted as described [29]. Prior to disruption, approximately a total of 2 × 10^7^ cells were suspended in 2 mL of lysis buffer consisting of 20 mM HEPES at pH 7.5, 10 mM KCl, 1.5 mM MgCl_2_, 1 mM Na-EDTA, 1 mM Na-EGTA, 1 mM DTT, 250 mM sucrose, and 0.1 % *v*/*v* protease inhibitor cocktail (2 mM AEBSF, 0.3 μM aprotinin, 130 μM bestatin, 14 mM E-64, 1 mM leupeptin, 1 mM EDTA). The HeLa cell extract (lysate) was stored at −80 °C for at least 5 days before use. Total protein content in the lysate was determined by the Bradford method. Each cyt c sample was incubated with the lysate in a total volume of 50 μL at 37 °C for 1 h using a Mastercycler (Eppendorf). This incubation mixture consisted of 1 mM dATP, 4 mg/mL total protein from lysate, and 10 μM cyt c; cyt c concentration was determined using the absorption coefficient 29.5 mM^−1^ cm^−1^ at 550 nm [32]. Immediately thereafter, caspase-3 activation was performed as per the manufacturer’s protocol (CaspACE™ Colorimetric Assay System, G7220). Briefly, 20 μL from the incubation mixture was added to 78 μL of a mixture containing 128.2 mM HEPES at pH 7.5, 12.82 % *w*/*v* sucrose, 0.1282 % *w*/*v* CHAPS, 2.56 % *v*/*v* DMSO, and 12.8 mM DTT. Afterwards, 2 μL of 10 mM caspase-3 substrate (Ac-DEVD-pNA) was added. The plate was incubated overnight, and the absorbance measured at 405 nm using a microplate reader (BioTek-Synergy H1 Hybrid Reader). All measurements were performed in triplicate. The activation by horse cyt c was considered 100 %, and other results were normalized relative to horse cyt c. In these assays, a negative control that contained HeLa cell lysate with asparaginase-II, an unrelated non-apoptotic protein, was used. Asparaginase-II was purified from *E. coli* as described earlier [33]. Additionally, an aqueous extract of *R. arrhizus* was also made and tested for caspase-3 activation (Additional file 5: Figure S6).

## Availability of supporting data

The data supporting the results of this article is included within the article in seven additional files.

## Additional files

Additional file 1: Figure S1. Mascot search results of the selected precursors of the recombinant and the native *R. arrhizus* cyt c peptides. (DOCX 18 kb) Additional file 2: Figure S2. Absorbance versus wavelength graph showing the charge transfer band of Rhizopus cyt c of both native and recombinant purified protein. (DOCX 80 kb) Additional file 3: Figure S3. Graph log of the ferro-/ferrocytochrome c (*R. arrhizus*) reaction quotients vs. log of the ferro/ferricyanide reaction quotients, used for redox potential calculation and the equation used for calculation. (DOCX 96 kb) Additional file 4: Figure S4. Western blot analysis to test the presence of cyt c in culture supernatants of *R. arrhizus* using horse cyt c monoclonal antibody. (DOCX 166 kb) Additional file 5: Figure S5. Caspase-3 activation assay showing comparison of activity of different cyt c and aqueous extract from *R. arrhizus* culture. (DOCX 106 kb) Additional file 6: Figure S6. MS/MS spectra of the recombinant Rhizopus cyt c for the peptide corresponding to K72 of yeast, to show absence of trimethylation. (DOCX 51 kb) Additional file 7: Figure S7. Vector map and the primers used in cloning. (DOCX 146 kb)

## Abbreviations

ABTS: 2,2′-azino-bis(3-ethylbenzothiazoline-6-sulphonic acid)
Apaf-1: Apoptotic protease activating factor 1
Cyt c: Cytochrome c
DTT: Dithiothreitol
MALDI MS: Matrix-assisted laser desorption/ionization mass spectroscopy
SDS-PAGE: Sodium dodecyl sulfate-polyacrylamide gel electrophoresis

## References

[1] Petrikkos G, Skiada A, Lortholary O, Roilides E, Walsh TJ, Kontoyiannis DP (2012). Epidemiology and clinical manifestations of mucormycosis. Clin Infect Dis.

[2] Mane RS, Watve JK, Mohite AA, Patil BC (2007). Rhinocerebral mucormycosis: a deadly disease on the rise. Indian J Otolaryngol.

[3] Shirazi F, Kontoyiannis DP (2013). Mitochondrial respiratory pathways inhibition in Rhizopus oryzae potentiates activity of posaconazole and itraconazole via apoptosis. Plos One.

[4] Pagano L, Valentini GC, Fianchi L, Caira M (2009). The role of neutrophils in the development and outcome of zygomycosis in haematological patients. Clin Microbiol Infec.

[5] Lamaignere CG, Simitsopoulou M, Roilides E, Maloukou A, MWinn R, Walsh TJ (2005). Interferon-γ and granulocyte-macrophage colony-stimulating factor augment the activity of polymorphonuclear leukocytes against medically important zygomycetes. J Infect Dis.

[6] Brust D, Hamann A, Osiewacz HD (2009). Apoptosis in fungal development and ageing. In Physiology and Genetics.

[7] Suzuki T, Ushikoshi S, Morita H, Fukuoka H (2007). Aqueous extracts of Rhizopus oryzae induce apoptosis in human promyelocytic leukemia cell line HL-60. J Health Sci.

[8] Kluck RM, Bossy-Wetzel E, Green DR, Newmeyer DD (1997). The release of cytochrome c from mitochondria: a primary site for Bcl-2 regulation of apoptosis. Science.

[9] Liu X, Kim CN, Yang J, Jemmerson R, Wang X (1996). Induction of apoptotic program in cell-free extracts: requirement for dATP and cytochrome c. Cell.

[10] Madeo F, Fröhlich E, Fröhlich KU (1997). A yeast mutant showing diagnostic markers of early and late apoptosis. J Cell Biol.

[11] Carmona-Gutierrez D, Eisenberg T, Büttner S, Meisinger C, Kroemer G, Madeo F (2010). Apoptosis in yeast: triggers, pathways, subroutines. Cell Death Differ.

[12] Silva RD, Sotoca R, Johansson B, Ludovico P, Sansonetty F, Silva MT (2005). Hyperosmotic stress induces metacaspase‐and mitochondria‐dependent apoptosis in Saccharomyces cerevisiae. Mol Microbial.

[13] Olteanu A, Patel CN, Dedmon MM, Kennedy S, Linhoff MW, Minder CM (2003). Stability and apoptotic activity of recombinant human cytochrome c. Biochem Biophys Res Commun.

[14] Patel CN, Lind MC, Pielak GJ (2001). Characterization of horse cytochrome c expressed in Escherichia coli. Protein Expres Purif.

[15] Obayashi A, Yorifuji H, Yamagata T, Ijichi T, Kanie M (1966). Respiration in organic acid-forming molds. Part I. Purification of cytochrome c, coenzyme Q9 and l-lactic dehydrogenase from lactate forming Rhizopus oryzae. Agric Biol Chem.

[16] Dolatabadi S, Hoog GS, Meis JF, Walther G (2014). Species boundaries and nomenclature of Rhizopus arrhizus (syn. R. oryzae). Mycoses.

[17] Janbon G, Rustchenko EP, Klug S, Scherer S, Sherman F (1997). Phylogenetic relationships of fungal cytochromes c. Yeast.

[18] Craig DB, Nichols ER (2006). Spectroscopic measurement of the redox potential of cytochrome c for the undergraduate biochemistry laboratory. J Chem Educ.

[19] Eddowes MJ, Hill HAO (1979). Electrochemistry of horse heart cytochrome c. J Am Chem Soc.

[20] Kagan VE, Tyurin VA, Jiang J, Tyurina YY, Ritov VB, Amoscato AA (2005). Cytochrome c acts as a cardiolipin oxygenase required for release of proapoptotic factors. Nat Chem Biol.

[21] Diederix REM, Ubbink M, Canters GW (2002). Peroxidase activity as a tool for studying the folding of c-type (50:50 v/v) cytochromes. Biochemistry.

[22] Kim NH, Jeong MS, Choi SY, Kang JH (2004). Peroxidase activity of cytochrome c. Bull Korean Chem Soc.

[23] Radi R, Thomson L, Rubbo H, Prodanov E (1991). Cytochrome c -catalyzed oxidation of organic molecules by hydrogen peroxide. Arch Biochem Biophys.

[24] Schnell S, Maini PK (2003). A century of enzyme kinetics: reliability of the KM and vmax estimates. Comm Theoret Biol.

[25] Yu T, Wang X, Purring-Koch C, Wei Y, McLendon GL (2001). A mutational epitope for cytochrome C binding to the apoptosis protease activation factor-1. J Biol Chem.

[26] Kluck RM, Ellerby LM, Ellerby HM, Naiem S, Yaffe MP, Margoliash E (2000). Determinants of cytochrome c pro-apoptotic activity, the role of lysine 72 trimethylation. J Biol Chem.

[27] Shingu-Vazquez M, Traven A (2011). Mitochondria and fungal pathogenesis: drug tolerance, virulence, and potential for antifungal therapy. Eukaryot Cell.

[28] Zhivotovsky B, Orrenius S, Brustugun OT, Døskeland SO (1998). Injected cytochrome c induces apoptosis. Nature.

[29] Méndez J, Morales-Cruz M, Delgado Y, Figueroa CM, Orellano EA, Morales M (2014). Deliver of chemically glycosylated cytochrome c immobilized in mesoporous silica nanoparticles induces apoptosis in HeLa cancer cells. Mol Pharm.

[30] Shevchenko A, Tomas H, Havlis J, Olsen JV, Mann M (2007). In-gel digestion for mass spectrometric characterization of proteins and proteomes. Nat Protoc.

[31] Kussmann M, Nordhoff E, Rahbek-Nielsen H, Haebel S, Rossel-Larsen M, Jakobsen L (1997). Matrix-assisted laser desorption/ionization mass spectrometry sample preparation techniques designed for various peptide and protein analytes. J of Mass Spectrom.

[32] Van Gelder BF, Slater EC (1962). The extinction coefficient of cytochrome c. Biochim Biophys Acta.

[33] Khushoo A, Pal Y, Singh BN, Mukherjee KJ (2004). Extracellular expression and single step purification of recombinant Escherichia coli L-asparaginase II. Prot Expr Purif.

