# Erratum to: Purification and characterization of a cytochrome c with novel caspase-3 activation activity from the pathogenic fungus *Rhizopus arrhizus*

**DOI:** 10.1186/s12858-016-0059-8

**Published:** 2016-02-19

**Authors:** Manoj Saxena, Rohit Kumar Sharma, Josell Ramirez-Paz, Arthur D. Tinoco, Kai Griebenow

**Affiliations:** Department of Environmental Sciences, University of Puerto Rico, Rio Piedras Campus, P.O. Box 70377, San Juan, PR 00936-837 USA; Department of Chemistry, University of Puerto Rico, Rio Piedras Campus, P.O. Box 70377, San Juan, PR 00936-837 USA

After publication of the original article [1], it was noticed that the links for Additional file 4: Figure S4, Additional file 5: Figure S5, Additional file 6: Figure S6 and Additional file 7: Figure S7 had been incorrectly reverted and therefore linked to the incorrect documents.

The correct linking for the Additional Files is included in this erratum.

## Additional files

Additional file 4: Figure S4. Western blot analysis to test the presence of cyt c in culture supernatants of *R. arrhizus* using horse cyt c monoclonal antibody. (DOCX 166 kb) Additional file 5: Figure S5. Caspase-3 activation assay showing comparison of activity of different cyt c and aqueous extract from *R. arrhizus* culture. (DOCX 106 kb) Additional file 6: Figure S6. MS/MS spectra of the recombinant Rhizopus cyt c for the peptide corresponding to K72 of yeast, to show absence of trimethylation. (DOCX 51 kb) Additional file 7: Figure S7. Vector map and the primers used in cloning. (DOCX 149 kb)
