# Supplementary material for: Purification and characterization of a cytochrome c with novel caspase-3 activation activity from the pathogenic fungus Rhizopus arrhizus
Source: BMC Biochem. 2015 Sep 3;16:21. doi: 10.1186/s12858-015-0050-9 (PMC4559206; doi:10.1186/s12858-015-0050-9)
Supplement: Additional file 1: Figure S1. — Mascot search results of the selected precursors of the recombinant and the native R. arrhizus cyt c peptides. (DOCX 18 kb) [file 12858_2015_50_MOESM1_ESM.docx]

A. MASCOT Search Result- Recombinant cytochrome c

Taxonomy- Rhizopus delemar RA 99-880

Enzyme- Trypsin, Database- NCBInr, Score-80

Protein sequence coverage- 53%

Matched peptides are shown in bold

1 MAEGNATAGA KLFKTRCAQC HTVEEGGANK **VGPNLHGIFG** **RKSGQVAGYD**

51 **YTAANKNKGV** **TWDEQTLFDY LENPKK**YIPG TKMAFAGFKK AK**DRSDVVAY**

101 **LK**DAC

Start-End Observed Mr (expt) Mr (Calc) Δ M

31-41 1166.8383 1165.8310 1165.6356 0.1954

42-56 1573.2250 1572.2177 1571.7580 0.4598

43-58 1687.3535 1686.3472 1685.8009 0.5463

59-76 2183.9758 2182.9685 2182.0582 0.9103

93-102 1165.7986 1164.7913 1164.6139 0.1174

B. MASCOT Search Result- Native cytochrome c

Taxonomy- Rhizopus delemar RA 99-880

Enzyme- Trypsin, Database- NCBInr, Score-161

Protein sequence coverage-24%

Matched peptide/s shown in bold

1 MAEGNATAGA KLFKTRCAQC HTVEEGGANK **VGPNLHGIFG** **RKSGQVAGYD**

51 **YTAANK**NKGV TWDEQTLFDY LENPKKYIPG TKMAFAGFKK AKDRSDVVAY

101 LKEAC

Start-End Observed Mr (expt) Mr (Calc) Δ M Score

31-41 1166.6776 1165.6703 1165.6356 0.0347 60

42-56 1572.7471 1571.7398 1571.7580 -0.0181 66

**Supplementary Figure 1.** Mascot search results of the selected precursors of the recombinant and the native *R. arrhizus* cyt c. After in gel trypsin digestion, peptides were fragmented by MALDI-MSMS and results were analyzed using the Mascot online server A) Database match for the recombinant protein peptides. B) Match for the purified native protein derived peptide.
