# Supplementary material for: Purification and characterization of a cytochrome c with novel caspase-3 activation activity from the pathogenic fungus Rhizopus arrhizus
Source: BMC Biochem. 2015 Sep 3;16:21. doi: 10.1186/s12858-015-0050-9 (PMC4559206; doi:10.1186/s12858-015-0050-9)
Supplement: Additional file 2: Figure S2. — Absorbance versus wavelength graph showing the charge transfer band of Rhizopus cyt c of both native and recombinant purified protein. (DOCX 80 kb) [file 12858_2015_50_MOESM2_ESM.docx]

**A B**


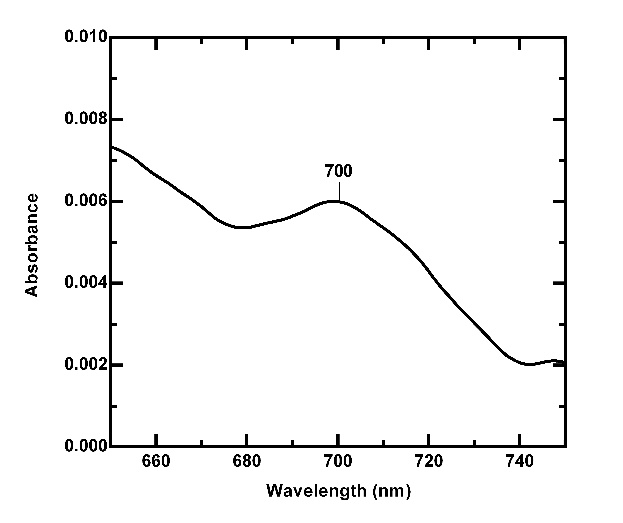

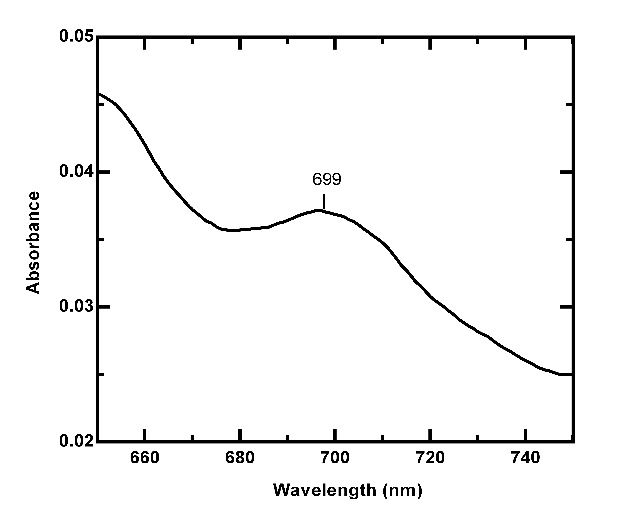


**Supplementary Figure 2.** The charge transfer band (CTB) of *R. arrhizus* cyt c with a maximum at around 700 nm. Peak locations were selected using the peak pick function of UVProbe software of UV-2450 Shimadzu spectrophotometer A) CTB in native cyt c from *R. arrhizus* B) CTB in the recombinant cyt c of *R. arrhizus.*
