# Supplementary material for: Purification and characterization of a cytochrome c with novel caspase-3 activation activity from the pathogenic fungus Rhizopus arrhizus
Source: BMC Biochem. 2015 Sep 3;16:21. doi: 10.1186/s12858-015-0050-9 (PMC4559206; doi:10.1186/s12858-015-0050-9)
Supplement: Additional file 3: Figure S3. — Graph log of the ferro-/ferrocytochrome c (R. arrhizus) reaction quotients vs. log of the ferro/ferricyanide reaction quotients, used for redox potential calculation and the equation used for calculation. (DOCX 96 kb) [file 12858_2015_50_MOESM3_ESM.docx]

**A**


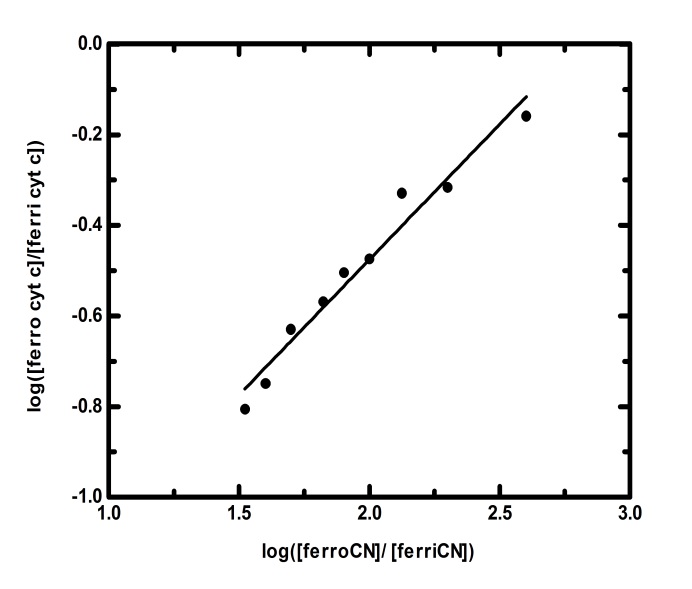

$$E= E^{\circ}-2.303 \frac{RT}{nf} log\left( \frac{\left[ ferroCN \right]}{\left[ ferriCN \right]} \right)$$

E = half-cell reduction potential at the temperature of interest

E
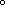
 = standard half-cell reduction potential

R = universal gas constant

T = absolute temperature

n = number of moles of electrons transferred in the balanced equation

f = faraday's constant

**B**

**
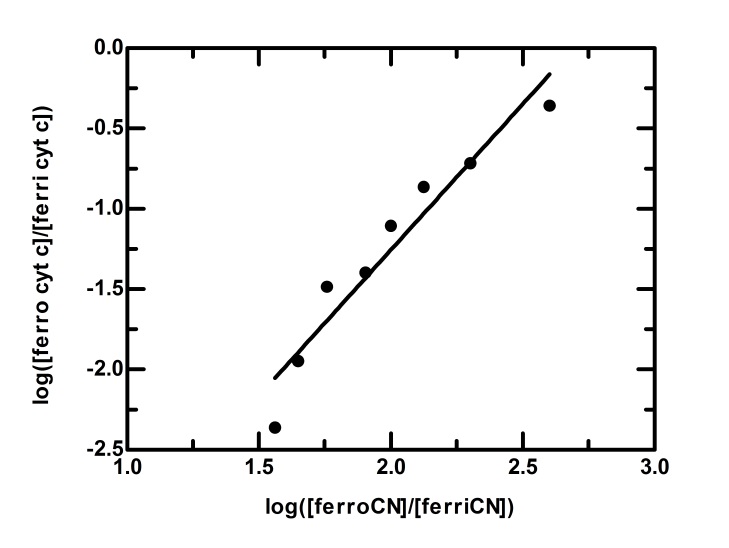
**

**Supplementary Figure 3.** Log of the ferro-/ferrocytochrome c (*R. arrhizus*) reaction quotients vs. log of the ferro-/ferricyanide reaction quotients, used for redox potential calculation. A. Native *R. arrhizus* cyt c. B. Recombinant *R. arrhizus* cyt c.
