# Supplementary material for: Purification and characterization of a cytochrome c with novel caspase-3 activation activity from the pathogenic fungus Rhizopus arrhizus
Source: BMC Biochem. 2015 Sep 3;16:21. doi: 10.1186/s12858-015-0050-9 (PMC4559206; doi:10.1186/s12858-015-0050-9)
Supplement: Additional file 4: Figure S4. — Western blot analysis to test the presence of cyt c in culture supernatants of R. arrhizus using horse cyt c monoclonal antibody. (DOCX 166 kb) [file 12858_2015_50_MOESM4_ESM.docx]

**
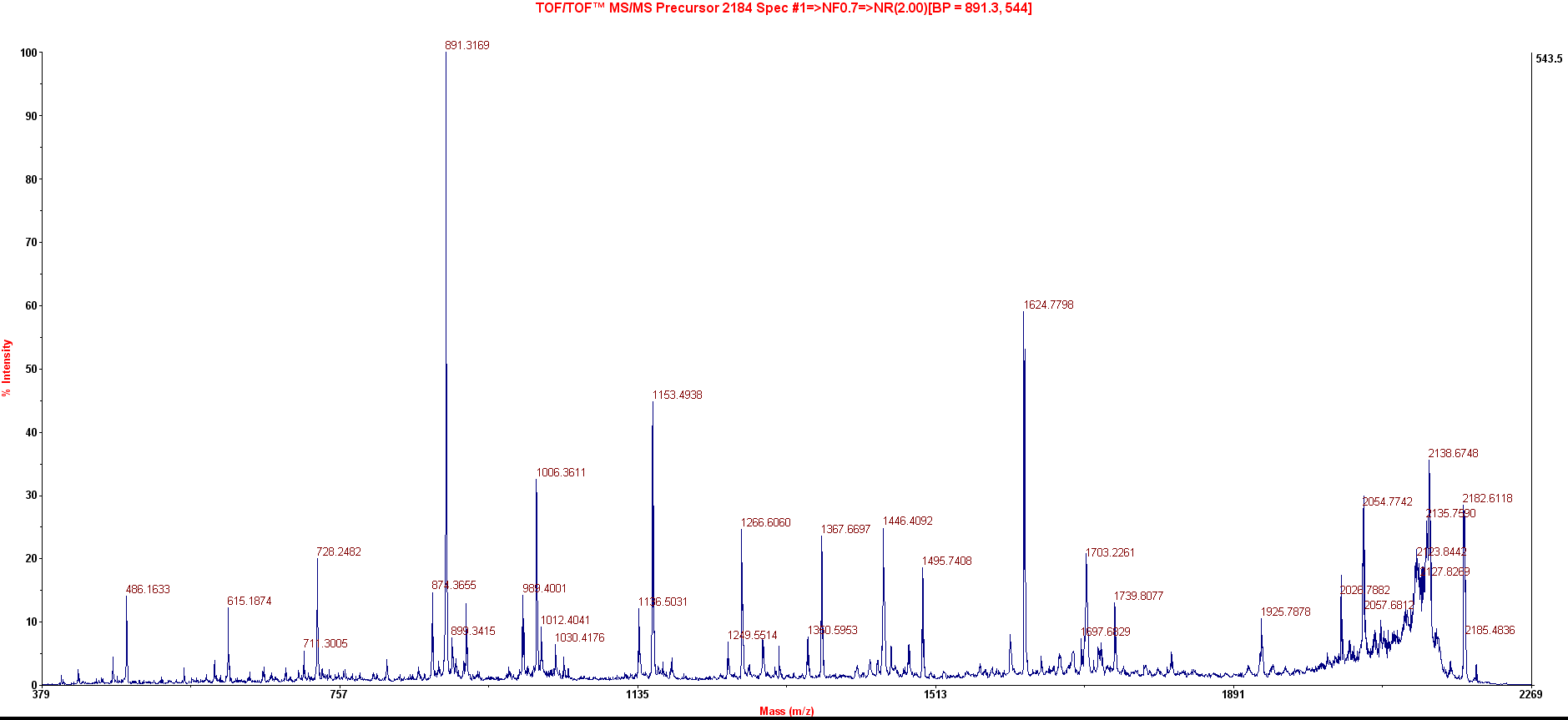
**

**Observed Mr(expt) Mr(calc) Delta Miss Score Expect Rank Unique Peptide**

**2184.0000 2182.9927 2182.0582 0.9345 1 86 2e-05 1 U K.GVTWDEQTLFDYLENPKK.Y**

**Supplementary Figure 4.** MS/MS spectra of a specific peptide to check possible tri-methylation at lysine corresponding to K-72 of budding yeast. Observed and theoretical mass of the peptide from recombinant *R. arrhizus* protein having the lysine corresponding to K-72 of yeast indicates the absence of tri-methylation of lysine in *R. arrhizus.* A tri-methylation would have increased the observed peptide mass by 42 Da (14*3).
