# Supplementary material for: Purification and characterization of a cytochrome c with novel caspase-3 activation activity from the pathogenic fungus Rhizopus arrhizus
Source: BMC Biochem. 2015 Sep 3;16:21. doi: 10.1186/s12858-015-0050-9 (PMC4559206; doi:10.1186/s12858-015-0050-9)
Supplement: Additional file 5: Figure S5. — Caspase-3 activation assay showing comparison of activity of different cyt c and aqueous extract from R. arrhizus culture. (DOCX 106 kb) [file 12858_2015_50_MOESM5_ESM.docx]

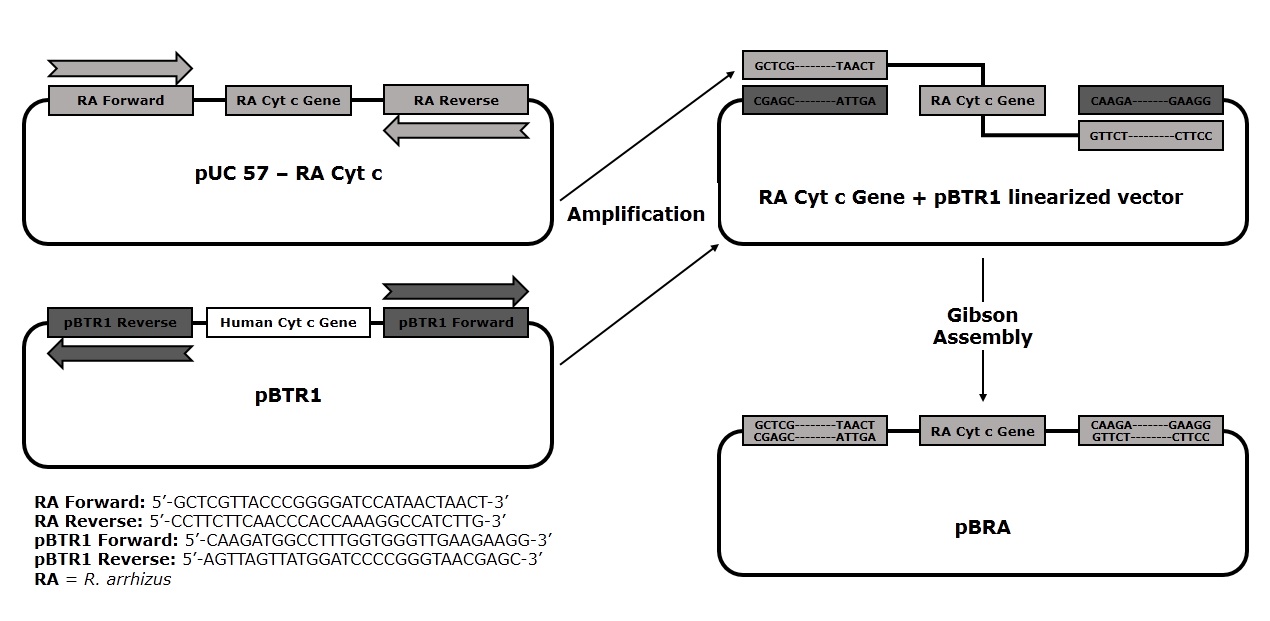


**Supplementary Figure 5.** The commercially synthesized *R. arrhizus* cyt c gene (in cloning vector pUC57) was amplified using the primers RA forward and RA reverse. The plasmid pBTR1 (containing human cyt c gene) was amplified using the primers pBTR1 forward and pBTR1 reverse, to produce the linearized pBTR1 vector (without human cyt c gene). Then, both fragments (with overlapping sequences) were incubated in a Gibson Assembly reaction to generate the pBRA plasmid with the *R. arrhizus* cyt c gene. *R. arrhizus* cyt c gene insertion was confirmed by commercial DNA sequencing (MCLAB).
