# Supplementary material for: Purification and characterization of a cytochrome c with novel caspase-3 activation activity from the pathogenic fungus Rhizopus arrhizus
Source: BMC Biochem. 2015 Sep 3;16:21. doi: 10.1186/s12858-015-0050-9 (PMC4559206; doi:10.1186/s12858-015-0050-9)
Supplement: Additional file 6: Figure S6. — MS/MS spectra of the recombinant Rhizopus cyt c for the peptide corresponding to K72 of yeast, to show absence of trimethylation. (DOCX 51 kb) [file 12858_2015_50_MOESM6_ESM.docx]

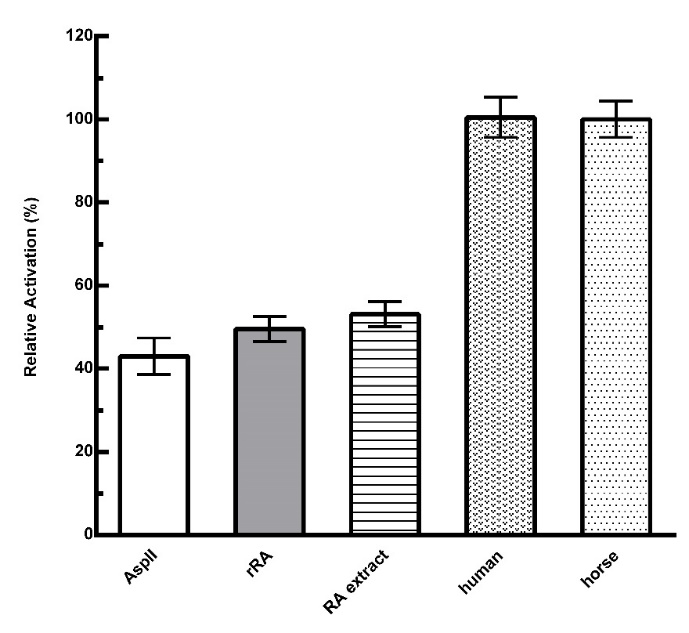


**Supplementary Figure 6.** Comparison of cell-free caspase-3 activation by horse, human and *R. arrhizus* cyt c. Caspase-3 activation was followed at 405 nm and normalized to the horse cyt c signal. rRA represents the recombinant cyt c from *R. arrhizus*, while RA extract is the 10x extract solution from R. *arrhizus* culture as detailed below (*Aqueous extract of R. arrhizus****)***, and AspII is the negative control L-asparaginase II. Each column represents the mean of independent measurements, with error bars representing the standard deviation. The initial incubation to activate caspase was performed with samples at 1.5 µM final concentration, except for the RA extract in which final concentration was 2x. The caspase-3 reaction was performed in 2 mM final substrate concentration (Sigma Caspase 3 Colorimetric Assay Kit); there were no statistically significant differences found between the signals of L-asparaginase II, recombinant *R*. *arrhizus* cyt c and its culture extract.

***Aqueous extract of R. arrhizus***

A 10 ml overnight starter culture of *R*. *arrhizus FGSC-9543 (obtained from-Fungal genetics Stock Center, University of Missouri, Kansas City)* grown in potato dextrose broth was used to inoculated 500 ml of potato dextrose broth and incubated for 24 h (25°C, 200 rpm) [1]. After incubation, fungi culture pellet was collected by centrifugation at 4°C and 7000 g for 30 min. Pellets were suspended in water and stirred for 1 hour at 45°C. The solution was then centrifuged at 4°C at 3000 g for 1 h, and the supernatant filtered **[1]**. The clear supernatant was concentrated using 3000 Da AMICON Ultrafiltration device until 10 times its original concentration. The concentrated (10x) *R*. *arrhizus* solution was tested in the caspase-3 activation assay.

References (additional data file 6)

1. Suzuki T, Ushikoshi S, Morita H, Fukuoka H. Aqueous extracts of Rhizopus oryzae induce apoptosis in human promyelocytic leukemia cell line HL-60. J Health Sci. 2007;53:760-5.
