# Supplementary material for: Purification and characterization of a cytochrome c with novel caspase-3 activation activity from the pathogenic fungus Rhizopus arrhizus
Source: BMC Biochem. 2015 Sep 3;16:21. doi: 10.1186/s12858-015-0050-9 (PMC4559206; doi:10.1186/s12858-015-0050-9)
Supplement: Additional file 7: Figure S7. — Vector map and the primers used in cloning. (DOCX 146 kb) [file 12858_2015_50_MOESM7_ESM.docx]

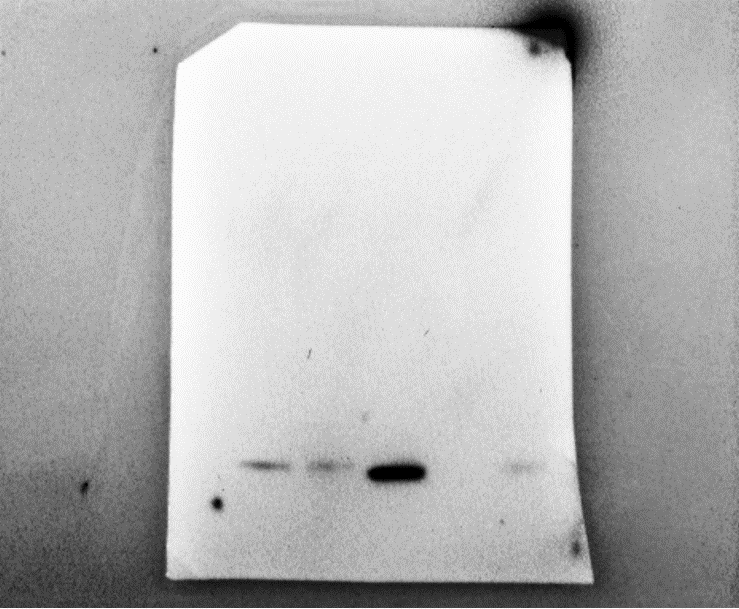


1 2

**Supplementary Fig.** 7 Western blot using horse cyt c monoclonal antibody. lane-1 concentrated supernatant of *R.arrhizus* culture, lane- 2 purified *R.arrhizus* recombinant cyt c.
