# Supplementary material for: Erratum to: Purification and characterization of a cytochrome c with novel caspase-3 activation activity from the pathogenic fungus Rhizopus arrhizus
Source: BMC Biochem. 2016 Feb 19;17:3. doi: 10.1186/s12858-016-0059-8 (PMC4761177; doi:10.1186/s12858-016-0059-8)
Supplement: Additional file 4: Figure S4. — Western blot analysis to test the presence of cyt c in culture supernatants of R. arrhizus using horse cyt c monoclonal antibody. (DOCX 166 kb) [file 12858_2016_59_MOESM1_ESM.docx]

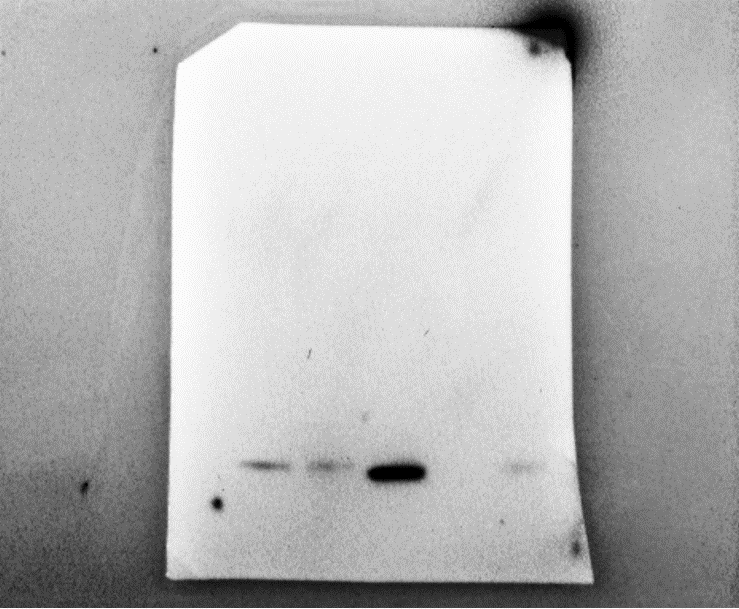


1 2

**Supplementary Figure.** **4**  Western blot using horse cyt c monoclonal antibody. lane-1 concentrated supernatant of *R.arrhizus* culture, lane- 2 purified *R.arrhizus* recombinant cyt c.
