# Supplementary material for: Erratum to: Purification and characterization of a cytochrome c with novel caspase-3 activation activity from the pathogenic fungus Rhizopus arrhizus
Source: BMC Biochem. 2016 Feb 19;17:3. doi: 10.1186/s12858-016-0059-8 (PMC4761177; doi:10.1186/s12858-016-0059-8)
Supplement: Additional file 6: Figure S6. — MS/MS spectra of the recombinant Rhizopus cyt c for the peptide corresponding to K72 of yeast, to show absence of trimethylation. (DOCX 51 kb) [file 12858_2016_59_MOESM3_ESM.docx]

**
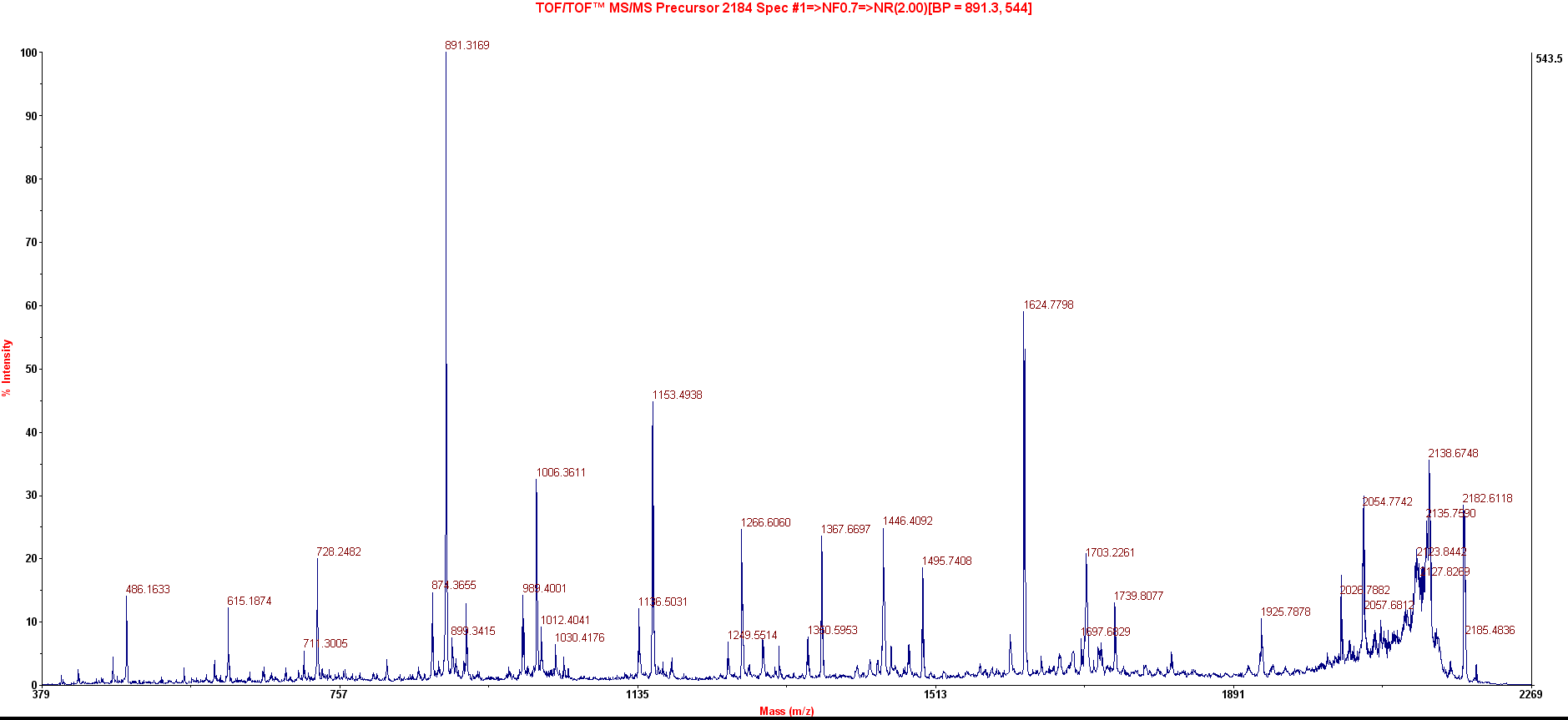
**

**Observed Mr(expt) Mr(calc) Delta Miss Score Expect Rank Unique Peptide**

**2184.0000 2182.9927 2182.0582 0.9345 1 86 2e-05 1 U K.GVTWDEQTLFDYLENPKK.Y**

**Supplementary Figure 6.** MS/MS spectra of a specific peptide to check possible tri-methylation at lysine corresponding to K-72 of budding yeast. Observed and theoretical mass of the peptide from recombinant *R. arrhizus* protein having the lysine corresponding to K-72 of yeast indicates the absence of tri-methylation of lysine in *R. arrhizus.* A tri-methylation would have increased the observed peptide mass by 42 Da (14*3).
