# Supplementary material for: Erratum to: Purification and characterization of a cytochrome c with novel caspase-3 activation activity from the pathogenic fungus Rhizopus arrhizus
Source: BMC Biochem. 2016 Feb 19;17:3. doi: 10.1186/s12858-016-0059-8 (PMC4761177; doi:10.1186/s12858-016-0059-8)
Supplement: Additional file 7: Figure S7. — Vector map and the primers used in cloning. (DOCX 149 kb) [file 12858_2016_59_MOESM4_ESM.docx]

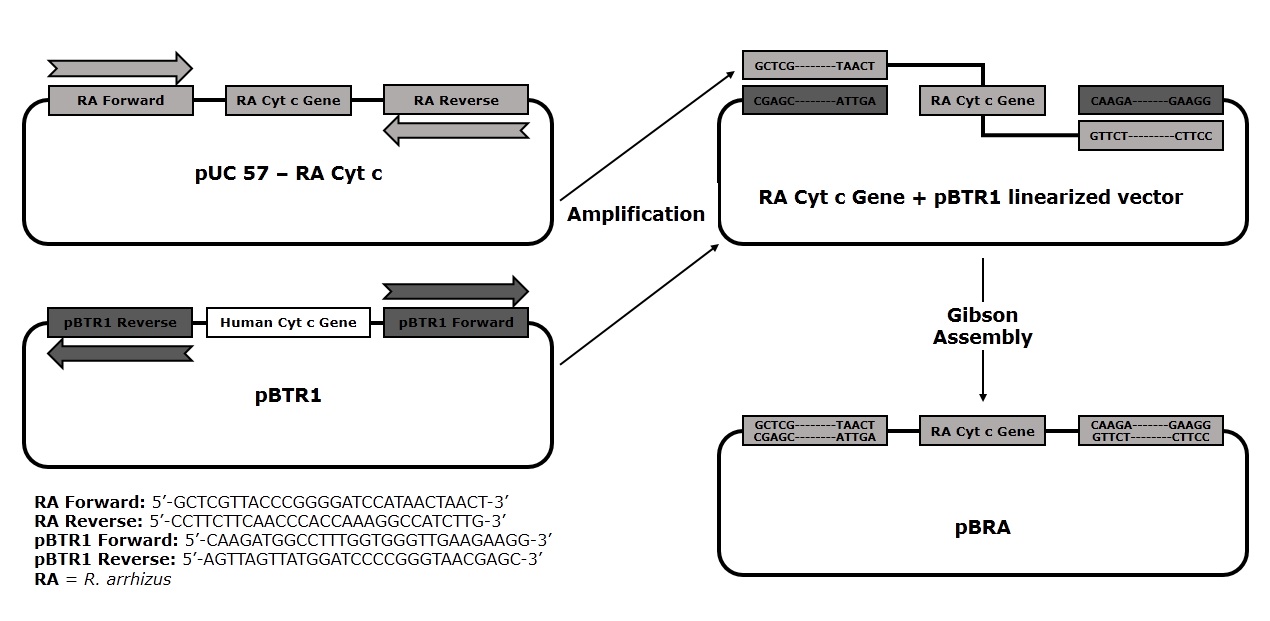


**Supplementary Figure 7.** The commercially synthesized *R. arrhizus* cyt c gene (in cloning vector pUC57) was amplified using the primers RA forward and RA reverse. The plasmid pBTR1 (containing human cyt c gene) was amplified using the primers pBTR1 forward and pBTR1 reverse, to produce the linearized pBTR1 vector (without human cyt c gene). Then, both fragments (with overlapping sequences) were incubated in a Gibson Assembly reaction to generate the pBRA plasmid with the *R. arrhizus* cyt c gene. *R. arrhizus* cyt c gene insertion was confirmed by commercial DNA sequencing (MCLAB).
